# Supplementary material for: Back reaction of the untwisting solar corona scars sunspots
Source: Sci Adv. 2026 Jul 23;12(30):eaed5294. doi: 10.1126/sciadv.aed5294 (PMC13394465; doi:10.1126/sciadv.aed5294)
Supplement: Supplementary file 1 — Supplementary Text Figs. S1 to S4 [file sciadv.aed5294_sm.pdf]

Science Advances
AAAS

Supplementary Materials for

Back reaction of the untwisting solar corona scars sunspots

Chen Xing *et al.*

Corresponding author: Chen Xing, chenxing@nju.edu.cn; Xin Cheng, xincheng@nju.edu.cn

Sci. Adv. **12**, eaed5294 (2026)
DOI: 10.1126/sciadv.aed5294

This PDF file includes:

Supplementary Text
Figs. S1 to S4

Supplementary Text

Relationship between Evolutions of Scar and Untwisting Coronal Loops

One can find that the period of the untwisting of twisted coronal loops in 131 Å and 304 Å images (08:00-10:12 UT; Fig. 2) is shorter than that of the sunspot scar growth (07:34-11:34 UT; Fig. 3M). Nevertheless, it does not substantially affect our result that the untwisting of coronal fields leads to the growth of sunspot scar, considering the following reasons. First, the untwisting process likely occurred over a longer period covering 08:00-10:12 UT, while it was only visible in AIA images during 08:00-10:12 UT when the temperature of twisted coronal loops was within the instrument's observation temperature range. In fact, the temperature of twisted coronal loops does vary, as they initially appeared in 131 Å and were subsequently observable only in 304 Å (Fig. 2). Second, the end of the sunspot scar growth should be later than that of the untwisting phenomenon in the corona, because it takes time for the latter to cause the former through the propagation of Alfvén waves whose speed is especially low in the lower atmosphere. Third, when Alfvén waves (launched by the untwisting of coronal loops) reach the positive-polarity footpoint of loops, a part of waves is refracted into the photosphere, causing a change of magnetic fields there and forming the sunspot scar. However, the other part of waves is reflected back into the corona and could propagate back to and grow the sunspot scar after reflected again at the other footpoint of loops. Such a possible process could occur several times as the overlying loops gradual relax toward a mechanical equilibrium, which may also explain why the sunspot scar continues to grow after the end of the untwisting of coronal loops.

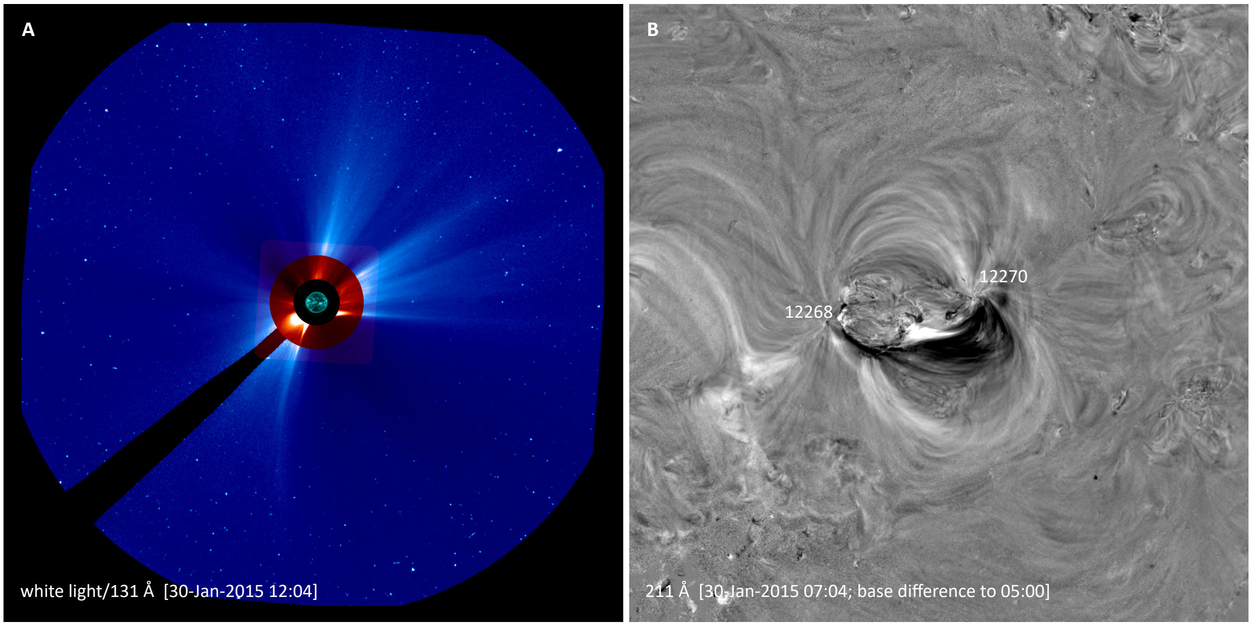

Figure S1: **Evidence for the failure of the eruption.** (**A**) Composite image of the white-light images of LASCO C2 and C3 and the AIA 131 Å image around 12:04 UT on 30 January 2015. (**B**) The base-difference image of the region around ARs 12268/70 in 211 Å at 07:04 UT on 30 January 2015, with the base image taken at 05:00 UT.

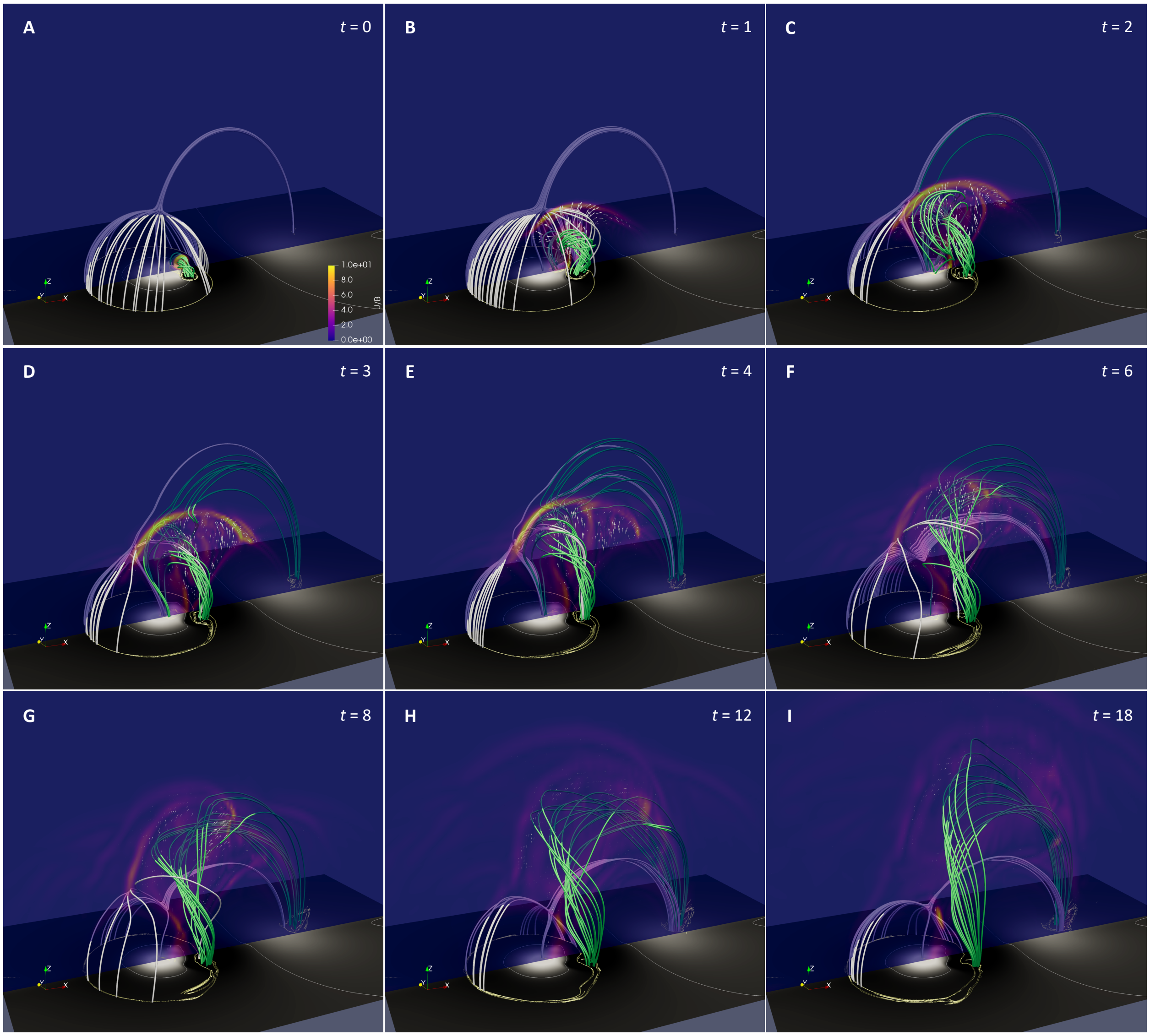

Figure S2: **Evolution of the modeled failed eruption.** The green field lines show the pre-eruptive/erupting structure. The white field lines, traced from the low field-strength region, represent the (remaining) fan-spine structure. The vertical surface, $y = 0$, shows the distribution of J/B. The white arrows represent the flow $v_x \boldsymbol{e}_x + v_z \boldsymbol{e}_z$ at this surface, with the length scales of arrows in each panel being the same. The bottom surface shows the distribution of B_z on the observation layer, where the yellow contours represent the boundaries of the footprints of QSLs and the white curves represent the PILs.

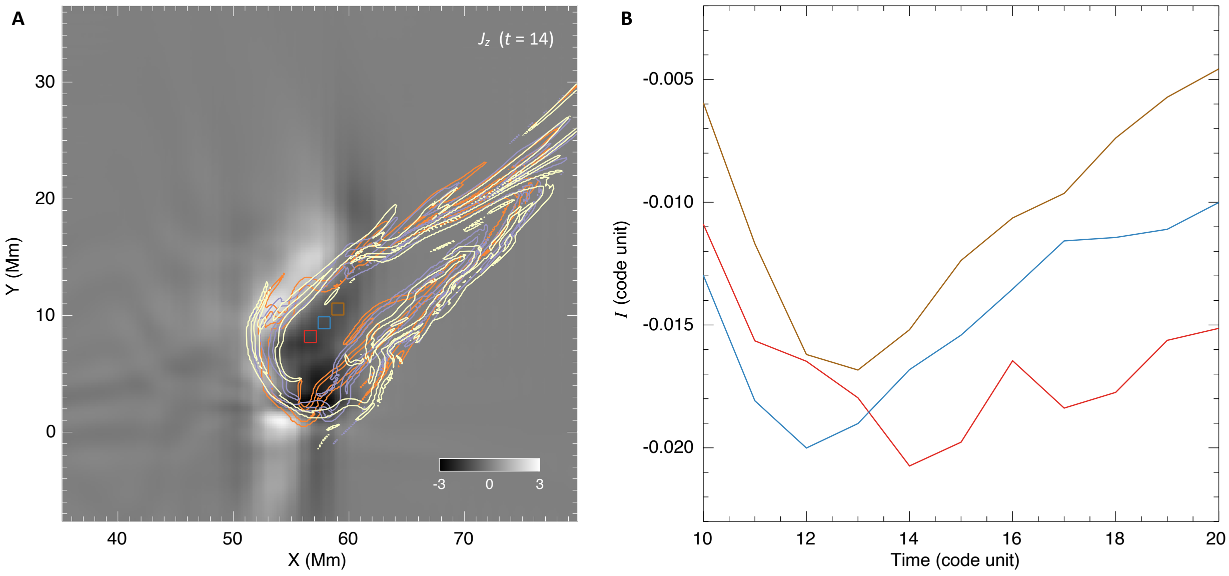

Figure S3: **Evolution of the current of the modeled sunspot scar.** (**A**) The distribution of J_z in the remote positive polarity on the observation layer at $t = 14$. The orange, purple, and yellow curves represent the boundaries of the footprints of QSLs at $t = 10$, $t = 14$, and $t = 20$, respectively. (**B**) The red, blue, and brown curves represent the evolutions of the currents (I) integrated in the red, blue, and brown boxes in panel A, respectively, the latter of which are always located in the negative-current region of sunspot scar during $10 \leq t \leq 20$.

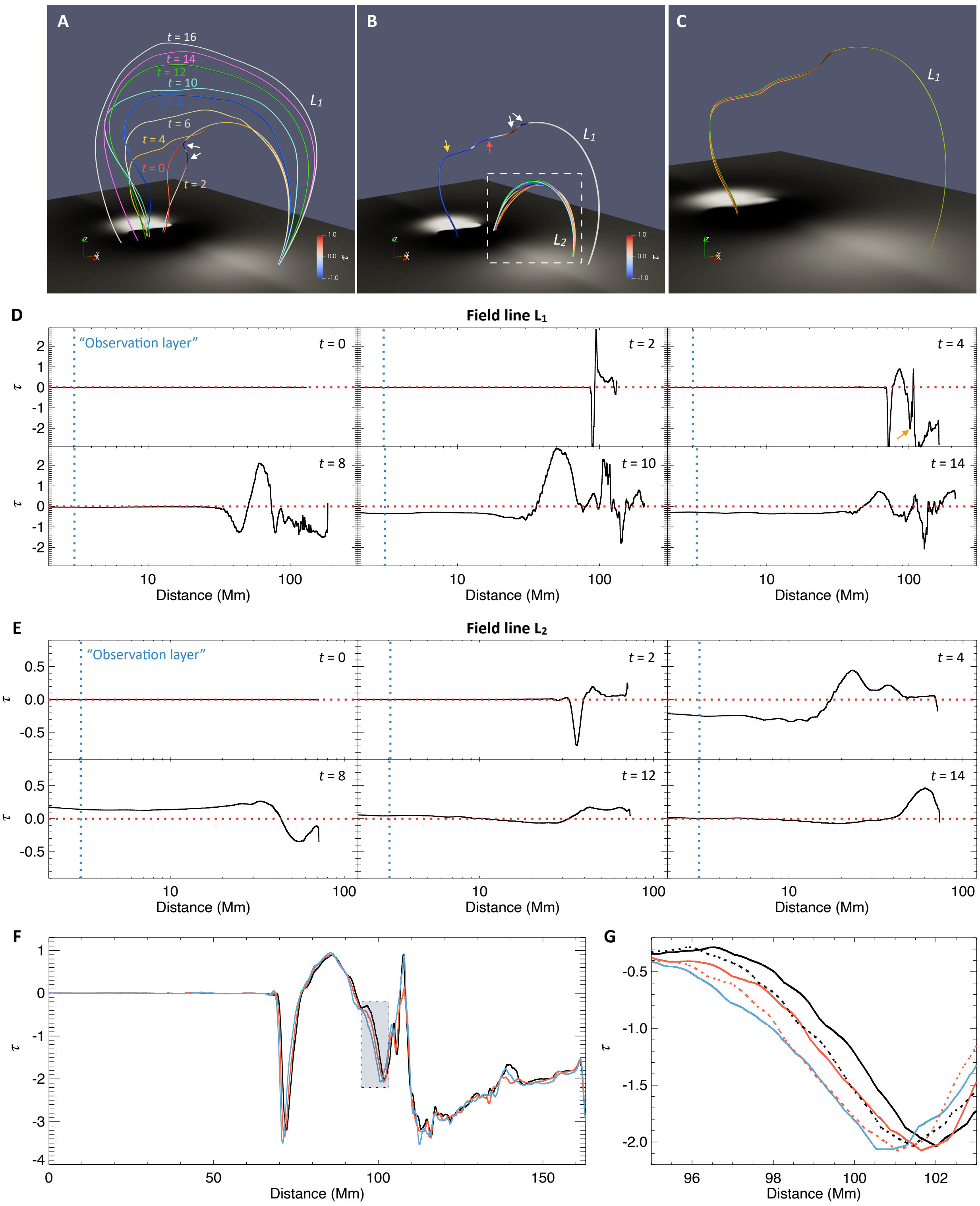

A
B
C
t = 16
t = 14
t = 12
t = 10
t = 8
t = 6
t = 4
t = 0
t = 2
L1
L2
D
Field line L1
"Observation layer"
t = 0
t = 2
t = 4
t = 8
t = 10
t = 14
τ
Distance (Mm)
E
Field line L2
"Observation layer"
t = 0
t = 2
t = 4
t = 8
t = 12
t = 14
τ
Distance (Mm)
F
τ
Distance (Mm)
G
τ
Distance (Mm)

Figure S4: **Evolutions of the field lines anchored around the sunspot scar.** (**A**) Evolution of L_1 during $0 \leq t \leq 16$. The color of the field line at $t = 2$ shows the distribution of τ along L_1, and the white arrows point to a pair of disturbance-induced twist. The bottom surfaces exhibit the distributions of B_z on the observation layer. (**B**) Similar to panel A but showing the evolution of L_2 (in the dashed box). In addition, the field line outside the dashed box represents L_1 at $t = 4$, and its color shows the distribution of τ along it. The white arrows point to the disturbance-induced twist. The yellow arrow points to the reconnection-induced twist, with the red arrow marking its leading front. (**C**) Evolution of L_1 from $t = 4$ (yellow) to $t = 4.05$ (red) to $t = 4.1$ (green). (**D**) Evolution of the distribution of τ along L_1. The distance refers to that along L_1 to its positive-polarity footpoint on the line-tied layer. The red dashed line marks $\tau = 0$, and the blue dashed line marks the intersection of L_1 and the observation layer. The yellow arrow points to the reconnection-induced twist. (**E**) Similar to panel D but showing the evolution of τ along L_2. (**F**) Evolution of the distribution of τ along L_1 from $t = 4$ (black) to $t = 4.05$ (red) to $t = 4.1$ (blue). (**G**) The zoom-in image of the gray region in panel F. The solid curves have the same meanings as those in panel F. The black (red) dashed curve represents the shifted distribution of τ at $t = 4.05$ ($t = 4.1$).
